# Nitrogen controls the net primary production of an alpine *Kobresia* meadow in the northern Qinghai‐Tibet Plateau

**DOI:** 10.1002/ece3.5442

**Published:** 2019-07-16

**Authors:** Licong Dai, Xun Ke, Yangong Du, Fawei Zhang, Yikang Li, Qian Li, Li Lin, Cuoji Peng, Kai Shu, Guangmin Cao, Xiaowei Guo

**Affiliations:** ^1^ Key Laboratory of Adaptation and Evolution of Plateau Botany, Northwest Institute of Plateau Biology Chinese Academy of Science Xining China; ^2^ University of Chinese Academy of Science Beijing China; ^3^ College of Life Sciences Luoyang Normal University Luoyang China

**Keywords:** aboveground net primary productivity, alpine *Kobresia* meadow, belowground net primary productivity, growing season air temperature, growing season precipitation

## Abstract

Net primary production (NPP) is a fundamental property of natural ecosystems. Understanding the temporal variations of NPP could provide new insights into the responses of communities to environmental factors. However, few studies based on long‐term field biomass measurements have directly addressed this subject in the unique environment of the Qinghai‐Tibet plateau (QTP). We examined the interannual variations of NPP during 2008–2015 by monitoring both aboveground net primary productivity (ANPP) and belowground net primary productivity (BNPP), and identified their relationships with environmental factors with the general linear model (GLM) and structural equation model (SEM). In addition, the interannual variation of root turnover and its controls were also investigated. The results show that the ANPP and BNPP increased by rates of 15.01 and 143.09 g/m^2^ per year during 2008–2015, respectively. BNPP was mainly affected by growing season air temperature (GST) and growing season precipitation (GSP) rather than mean annual air temperature (MAT) or mean annual precipitation (MAP), while ANPP was only controlled by GST. In addition, available nitrogen (AN) was significantly positively associated with BNPP and ANPP. Root turnover rate averaged 30%/year, increased with soil depth, and was largely controlled by GST. Our results suggest that alpine *Kobresia* meadow was an N‐limited ecosystem, and the NPP on the QTP might increase further in the future in the context of global warming and nitrogen deposition.

## 1 INTRODUCTION

Net primary productivity (NPP) is a vital component of the global carbon cycle and a fundamental property of terrestrial ecosystems. Studies of the temporal variations in NPP can greatly improve our understanding of biosphere–atmosphere interaction, of the terrestrial carbon cycle, and of how terrestrial ecosystems respond to climate change (Cramer et al., 1999). To date, very little work has explored the temporal variability of NPP based on long‐term field biomass measurements, which impedes the validation and evaluation of global carbon models (Cramer et al., 1999; Scurlock & Hall, 1998). Therefore, examining the interannual dynamics of NPP will contribute greatly to understanding how plants respond to external environmental factors in the context of global climate change (Knapp & Smith, 2001; Oesterheld, Loreti, Semmartin, & Sala, 2001); it will also aid predictions of likely ecosystem response to climate change (La Pierre et al., 2011). While the responses of aboveground productivity (ANPP) to climate variations have been well documented, the temporal variability of BNPP and its controlling environmental factors have received little attention due to the difficulty in measuring root biomass, yet BNPP accounts for more than half of total primary production in grasslands, and is also the major carbon stock (Luo, Sherry, Zhou, & Wan, 2009; Scurlock & Hall, 1998). Thus, understanding the temporal dynamics of NPP in terms of both BNPP and ANPP and their relationships with climate change factors could allow an improved assessment of terrestrial C budgets (Field, Behrenfeld, Randerson, & Falkowski, 1998; Piao, Fang, & He, 2006).

Grassland is widely distributed across arid and semi‐arid regions, and is of great importance in global carbon cycle (Yuanhe, Jingyun, Chengjun, & Wenxuan, 2009). Moreover, the grassland has shown great sensitivity to external climate factors such as changing precipitation and temperature. Consequently, a large number of studies have explored the temporal variability of grassland production and its controlling factors (Niklaus, Leadley, Schmid, & Körner, 2001; Nippert, Knapp, & Briggs, 2006), and many methods have been established to examine the response of NPP to climate change: These include long‐term monitoring, ecological modeling, and controlled experiments (Gao et al., 2009; Piao et al., 2006). Despite these efforts, general responses of NPP to climate change have not yet been reached regarding the key controlling factors that affect the grassland NPP, due to the complex interactions between environmental conditions as well as herbivorous animal populations and plant community composition (Zhang, Lal, Zhao, Jiang, & Chen, 2017). Furthermore, the response of NPP to environmental factors also varies with grassland types (Zhang et al., 2018). For instance, the NPP in arid and semi‐arid ecosystems was mainly limited by water (Oesterheld et al., 2001), while the NPP in a moist ecosystems was limited by temperature (Elmendorf et al., 2012). Therefore, it was necessary to obtain long‐term field measurements from a given study site to gain better understanding of the response mechanisms of alpine grassland to environmental factors.

Alpine grasslands are a widespread vegetation type at high altitudes. Alpine ecosystems are more limited by N limitations when compared with other ecosystems, owing to the extreme environmental conditions such as low temperature and shorter growing season (Gao et al., 2009). To date, there are great number of studies have been conducted regarding the net primary production in Tibetan alpine grasslands (Ma et al., 2017; Wu et al., 2011; Yuanhe et al., 2009). However, most previous studies have only focused on spatial scale, leaving a poor understanding on the temporal scale, due to their harsh climatic conditions. In addition, the root turnover in alpine ecosystems also plays an important role in the ecosystem nutrient dynamics, acting as an important sink of grassland productivity; the turnover ratio of roots follows a decreasing trend from tropical to high‐latitude systems (Gill & Jackson, 2000). Thus, a long‐term study on root turnover is necessary to better predict changes in ecosystem nutrient dynamics in natural ecosystems.

The Qinghai‐Tibet Plateau (QTP) is recognized as the world's highest and largest plateau. Alpine meadow and alpine grassland are the major land covers across the QTP (Yuanhe et al., 2009), and this ecosystem is more susceptible to climate change than that of other ecosystems. The region's low level of human disturbance, together with its unique geography, provides an ideal opportunity to examine the interannual dynamics of alpine grasslands and their relationship with environmental factors. Specifically, the objectives of this study are to (a) explore the interannual dynamics of ANPP and BNPP and their key controlling factors and (b) examine the interannual dynamics of turnover rate and its relationship with climate factors, particularly focus on the patterns of root turnover with depth, based on grassland productivity and environmental data collected during 2008–2015, and we hypothesized that the effects of air temperature on grassland productivity were mainly via altering soil available nitrogen.

## 2 MATERIALS AND METHODS

### 2.1 Study area

The study was conducted at Haibei National Field Research Station (37°37′N, 101°19′E, 3,200 m), located on the northern Tibetan Plateau, where the climate is characterized by a typical plateau continental monsoon climate. The mean annual air temperature is 1.7°C, and there are only two seasons (winter and summer). Winter is cold and dry with an average temperature of −14.8°C, and the summer is warm and rainy with an average temperature of 9.8°C. The average annual precipitation is approximately 580 mm, and almost 80% occurs in growing season (i.e., from May to September). The vegetation type at our study site is *Kobresia humilis* meadow, and the dominant species are *Kobresia humilis*. The study site was protected by fence from 2007, to prevent disturbance from human or grazing activities. The soil is classified as Mat‐cryosod soil, with a texture belonging to a loamy soil, and abundant organic matter in the top soil layer (approximately 12.7% in the top 0–10 cm); consequently, the soil has strong water‐holding capacity (Dai, Guo, Du, et al., 2019; Dai, Guo, Zhang, et al., 2019). The dimensions of this study site were 250 m × 230 m, covering typical alpine *Kobresia humilis* meadow. The relative abundances of grass, sedge, and forbs, by area, were 39.76%, 11.81%, and 48.43%, respectively.

### 2.2 Data collection

The belowground biomass (BGB) and aboveground biomass (AGB) (not include litter) were measured monthly during growing season (i.e., from May to September) in alpine *Kobresia* meadow from 2008 to 2015. AGB was obtained by the standard harvesting method in 10 randomly harvested quadrats (50 cm × 50 cm) within our study site (250 m × 230 m); the green and stand‐dead material was separated in aboveground biomass and was sorted into three plant functional groups: sedges, grasses, and forbs. The BGB data were sampled by extracting soil cores (diameter 7 cm) within 10 randomly harvested quadrats at depths of 0–10, 10–20, 20–30, and 30–40 cm on the basis that over 93% of root biomass is concentrated in the top 40 cm of soil (Cao, Du, Wang, Wang, & Liang, 2007), with five duplications, and then, the roots were washed carefully in sieves (0.5 mm) to remove the gravel. Finally, both AGB and BGB samples were oven‐dried at 65°C until reaching constant mass. In this study, the annual peak biomass (usually in August or early September) was adopted as the ANPP for each year, while BNPP was obtained by the max‐min method (Singh & Yadava, 1974), that is, the maximum BGB minus minimum BGB of each year. It should be noted that the BNPP may be overestimation due to the few dead roots were difficult to distinguish just based on only their color and consistency.

Given the little seasonal variation for soil nutrient properties but large interannual variation in our study site (Dai, Ke, et al., 2019; Lin et al., 2015), thus we obtain the soil chemical properties (available phosphorus, available nitrogen, total nitrogen content, and soil organic matter) only once in August each year from 2008 to 2015. And the soil sampling method was using the same method as that for BGB samples, that is, at depths of 0–10, 10–20, 20–30, and 30–40 cm soil profile among 10 randomly harvested quadrats. The samples collected as described above were sieved (2‐mm mesh) and then air‐dried before chemical analysis for their available phosphorus content (AP), available nitrogen content (AN), total nitrogen content (TN), and soil organic matter (SOM). The SOM was measured by wet dichromate oxidation of a homogenized air‐dried soil subsample (0.2 g), then titration with FeSO_4_. The AN and TN were determined by the Kjeldahl method and Smartchem 140, respectively. The AP measurement followed previous study (Verma et al., 2017).

Climatic variables used in this study include mean annual precipitation (MAP), growing season precipitation (GSP), mean annual air temperature (MAT), and growing season temperature (GST). The climate data were collected from the meteorological station at our study site.

### 2.3 Root turnover estimates

The root turnover of belowground biomass was determined after slightly modifying the model initially proposed by Dahlman and Kucera (1965). The root turnover is annual belowground production/mean belowground standing crop according to Dahlman and Kucera (1965) model. In this study, we substitute the mean belowground standing crop with maximum belowground standing crop, the formula as follows:

Root turnover=annual belowground production/maximum belowground standing crop,with root turnover in units of%/year.

### 2.4 Data analysis

The relationships between NPP (ANPP and BNPP), root turnover, and environmental factors were examined by a general linear model (GLM) and nonlinear function. To further understand the controls of ANPP and BNPP, the structural equation model (SEM) was applied to reveal the direct and indirect effect of climate variables on NPP. The main environmental factors such as GST and AN were included in the model. Firstly, we applied a full model to consider all possible pathways and then sequentially remove some insignificant pathways to obtain the best model. The model was improved by using modification indices, typically with the thresholds of modification indices was often set to 4.0. To evaluate the performance of the model, the Tucker–Lewis index, comparative fit index, and root mean square error of approximation were adopted in model evaluation calculated. The chi‐square test and unbiased maximum likelihood method were used in model identification and parameter estimation. The SEM was applied by the Lavaan package in R 3.33 (R Development Core Team, 2006). The general linear and nonlinear function was fitted by OrginPro 2015 (OriginLab).

## 3 RESULTS

### 3.1 Interannual dynamics of climate factors

**Figure 1:**
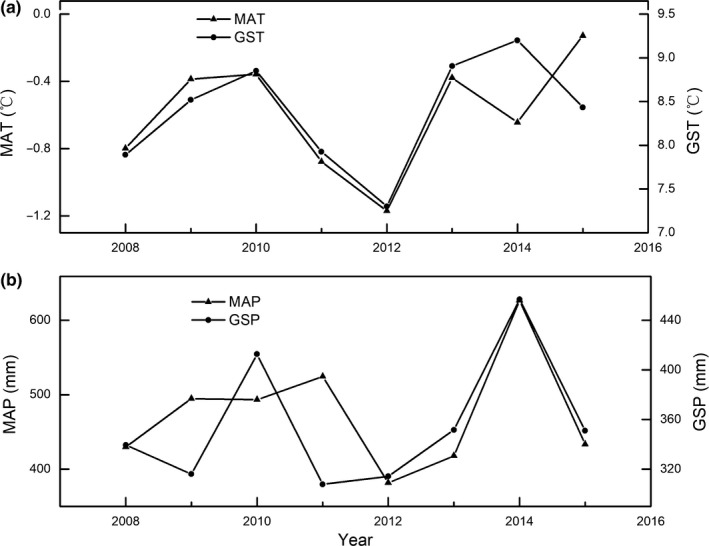
Interannual variations of temperature (a) and precipitation (b). GSP, growing season precipitation; GST, growing season temperature; MAP, mean annual precipitation; MAT, mean annual air temperature. The same below

Interannual fluctuation of both MAT and GST shows similar trends, that is, slightly increasing from 2008 to 2015 (*p* = 0.53 for MAT; *p* = 0.45 for GST) (Figure 1a). Interannual fluctuation in MAP was relatively small, whereas GSP showed a slightly increasing trend from 2008 to 2015 when compared with MAP, but this was not significant (*p* > 0.05; Figure 1b).

### 3.2 Interannual dynamics of ANPP, BNPP and root turnover

**Figure 2:**
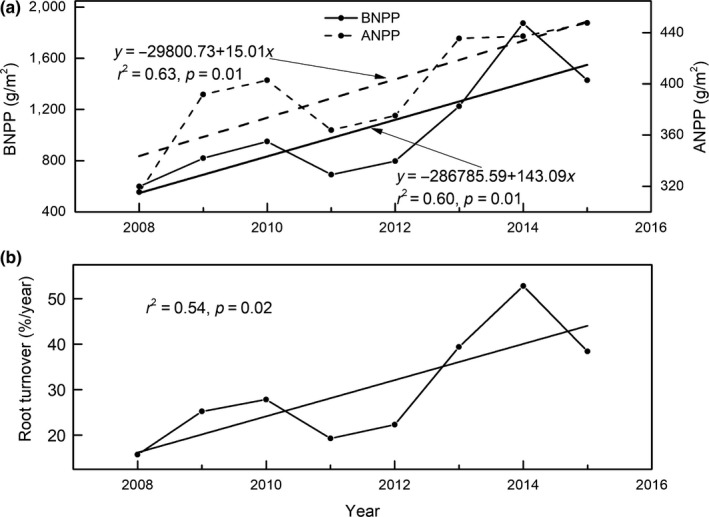
Interannual variations in aboveground net primary productivity (ANPP), belowground net primary productivity (BNPP) (a) and root turnover (b)

Both ANPP and BNPP followed increasing trends from 2008 to 2016, at rates of 15.01 g/m^2^ per year for ANPP and 143.09 g/m^2^ per year for BNPP (Figure 2a). The trend in ANPP was not significant (*p* > 0.05; Figure 2a). The averages of ANPP and BNPP during 2008–2015 were 396.14 g/m^2^ per year and 1,047.59 g/m^2^ per year, respectively. BNPP accounted for 72.91% of NPP (Tables 1 and 2). The root turnover exhibited a significantly increasing trend (Figure 2b; *p* < 0.05).

**Table 1:** Aboveground net primary productivity (ANPP) and monthly aboveground biomass (AGB) during the growing season from May to September 2008–2015

Year	ANPP (g/m^2^)	AGB‐May (g/m^2^)	AGB‐June (g/m^2^)	AGB‐July (g/m^2^)	AGB‐August (g/m^2^)	AGB‐September (g/m^2^)
2008	315.50	96.68	226.74	271.50	315.50	304.16
2009	391.69	70.56	140.38	312.77	391.69	294.20
2010	402.73	111.55	217.22	342.77	402.73	283.72
2011	363.86	76.62	229.15	330.30	363.86	362.97
2012	375.20	106.67	196.96	373.28	375.20	340.16
2013	435.52	58.08	181.92	350.40	435.52	339.04
2014	437.12	33.07	202.21	313.25	437.12	376.61
2015	447.52	69.44	166.72	327.36	393.28	447.52
Mean	396.14	77.83	195.16	327.70	389.36	343.55

**Table 2:** Belowground net primary productivity (BNPP) and monthly belowground biomass (BGB) during the growing season from May to September 2008–2015

Year	BNPP (g/m^2^)	BGB‐May (g/m^2^)	BGB‐June (g/m^2^)	BGB‐July (g/m^2^)	BGB‐August (g/m^2^)	BGB‐September (g/m^2^)
2008	597.95	3,704.80	3,802.50	3,204.55	3,265.28	3,214.16
2009	819.02	2,695.80	2,856.45	3,026.99	2,428.14	3,247.16
2010	949.29	2,460.64	2,677.86	2,815.54	2,853.28	3,409.93
2011	690.52	2,889.47	3,287.47	3,579.99	3,389.91	3,029.67
2012	796.99	2,842.47	3,295.92	2,774.85	2,945.87	3,571.83
2013	1,225.05	2,230.97	3,109.15	2,921.78	1884.10	2,413.36
2014	1873.15	2,800.31	2,765.05	2,957.36	1674.94	3,548.10
2015	1,428.73	2,701.99	2,291.36	3,147.32	2,374.94	3,720.10
Mean	1,047.59	2,790.81	3,010.72	3,053.55	2,602.057	3,269.29

### 3.3 Root turnover and BNPP across different soil layers

Belowground net primary productivity decreased with soil depth, with almost 64.81% of total BNPP distributed in the 0–10 cm layer while the 10–20, 20–30, and 30–40 cm layers only accounted for 15.62%, 14.57%, and 5%, respectively. In contrast, the mean turnover rate in the upper 0–40 cm was 30%/year and increased with soil depth: The maximum turnover rate occurred at 30–40 cm (41%/year), followed by 20–30 (40%/year), 10–20 (36%/year), and 0–10 cm (26%/year) (Table 3).

**Table 3:** Average of belowground net primary productivity (BNPP) and root turnover across different soil layers from 2008 to 2015

Soil depth (cm)	Maximum BGB (g/m^2^)	Minimum BGB (g/m^2^)	BNPP (g/m^2^)	Turnover (%/year)
0–10	2,571.26	1892.39	678.87	26
10–20	452.21	288.60	163.61	36
20–30	219.84	129.91	89.93	40
30–40	126.82	74.77	52.05	41
0–40	3,498.59	2,451.01	1,047.58	30

### 3.4 Dominant factors affecting the interannual variation of ANPP, BNPP, and root turnover

**Figure 3:**
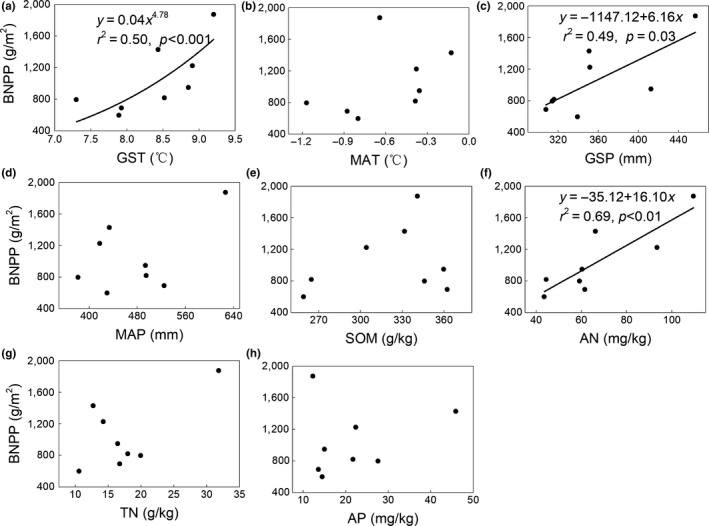
Relationships between environmental factors and belowground net primary productivity (BNPP)

**Figure 4:**
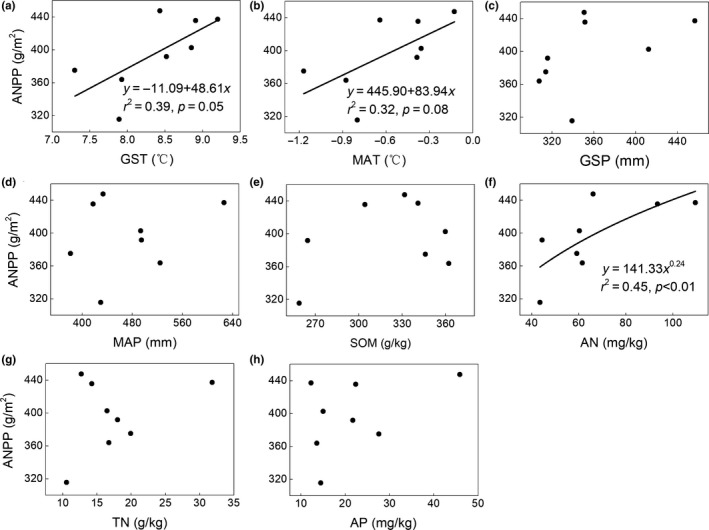
Relationships between environmental factors and aboveground net primary productivity (ANPP)

**Figure 5:**
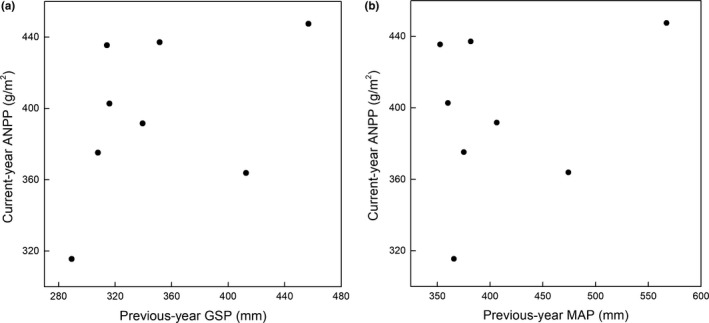
Relationship between previous‐year growing season precipitation (GSP), previous‐year mean annual precipitation (MAP) and current‐year ANPP

**Figure 6:**
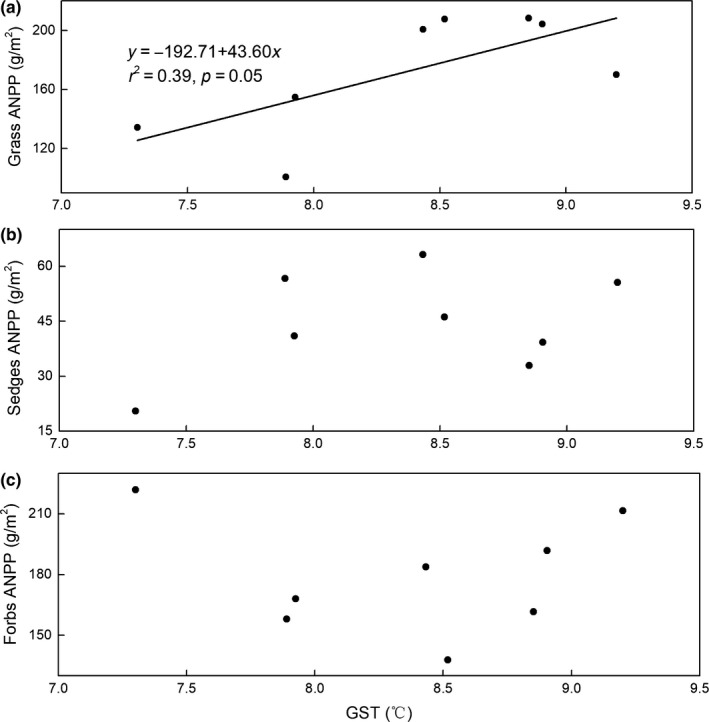
Relationships between growing season air temperature (GST) and different functional groups

**Figure 7:**
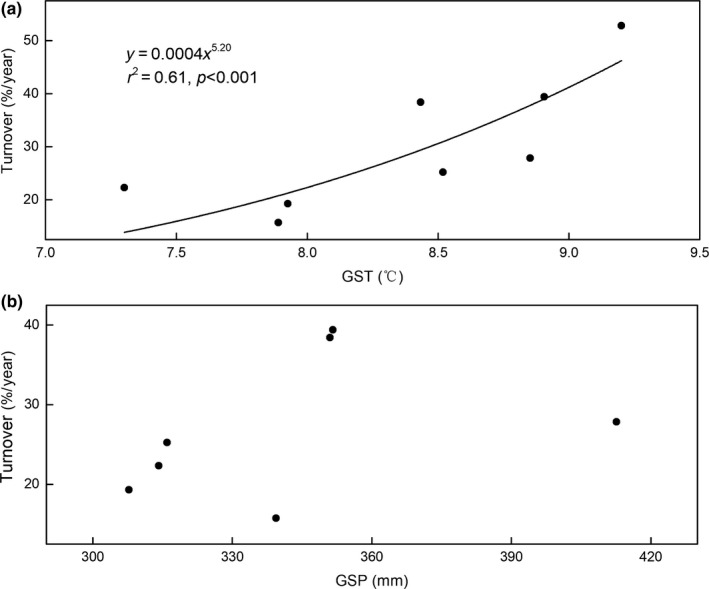
Relationships between growing season air temperature (GST), growing season precipitation (GSP) and root turnover

**Figure 8:**
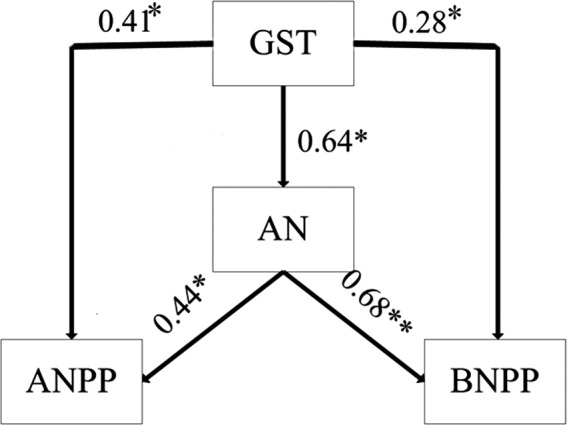
Structural equation models for the GST, AN, ANPP, and BNPP. The “**” and “*” represent *p* < 0.01 and *p* < 0.05, respectively. Chi‐square = 2.861 (*N* = 8, *p* = 0.09), CFI = 0.89, RMSEA = 0.09). Note: CFI = comparative fit index, RMSEA = root mean square error of approximation, the standardized path coefficients represent the effect size of the relationship, the model only show the statistically significant (*p* < 0.05) relationships

The general linear model indicated that both GST and GSP were significantly positively correlated with BNPP (*r* ^2^ = 0.50, *p* < 0.01 for GST; *r* ^2^ = 0.49, *p* = 0.03 for GSP; Figure 3a,c), whereas MAT and MAP had no significant effect on BNPP (*p* > 0.05; Figure 3b,d). For ANPP, the GST was significantly positively correlated with ANPP (Figure 4a), whereas both current‐year GSP and previous‐year GSP were not correlated with ANPP (*p* > 0.05; Figures 4c and 5). To better understand the relationship between ANPP and temperature, we then further examined the responses of ANPP within each functional group to GST and show that the grass ANPP was significantly positively related to GST (Figure 6a), whereas sedges and forbs ANPP were not correlated with GST (*p* > 0.05; Figure 6b,c). In addition, both ANPP and BNPP were significantly affected by AN (Figures 3f and 4f). The root turnover was significant correlations with GST (*r* ^2^ = 0.61, *p* < 0.001), while the GSP exert week impact on the root turnover (Figure 7). Overall, the GST can affect ANPP and BNPP via direct effect and indirect effect by altering AN (Figure 8).

## 4 DISCUSSION

### 4.1 Dominant factors affecting the interannual variation of ANPP and BNPP

It has been well documented that temperature and precipitation play an important role in grassland productivity (Liu et al., 2018; Sun, Cheng, & Li, 2013; Zhang et al., 2017).

Several studies have indicated that current‐year or previous‐year precipitation was dominant factors influencing the spatial or temporal patterns of ANPP (Bai, Han, Wu, Chen, & Li, 2004; Landesman & Dighton, 2010). For instance, ANPP is primarily controlled by moisture in Inner Mongolia (Bai et al., 2004; Landesman & Dighton, 2010) and in semi‐arid grassland (Oesterheld et al., 2001). In contrast, the ANPP in our study site was affected by temperature, and not controlled by either current‐ or previous‐year precipitation (Figure 4). Moreover, the ANPP was only determined by growing season temperature (GST) and not by mean annual air temperature (MAT). Similar results were also reported from temperate grasslands in North America (Knapp & Smith, 2001), arctic ecosystems (Schäppi, 1996), and other alpine meadows (Wielgolaski & Karlsen, 2007). Although ANPP was positively related to precipitation at large scales (Sala, Parton, Joyce, & Lauenroth, 1988), caution should be used when considering the differences in climate conditions of individual sites. For instance, the plants in our alpine ecosystem were limited by the low temperature and short growing season (Dai, Ke, et al., 2019). Moreover, the soil moisture was relative abundance relatively abundant during the growing season due to the replenishment from precipitation and thawing of seasonally frozen soil (Dai et al., 2018). Meanwhile, the soil in our study site was belonged to loamy soil with abundance abundant SOM in the top soil, lead to yielding a strong water‐holding capacity (Zhang et al., 2018). Thus, the ANPP was more sensitive to fluctuations in temperature (particularly growing season temperature) when compared with precipitation (Wielgolaski & Karlsen, 2007). Warmer temperatures can promote grassland productivity via two processes. Firstly, increased temperatures can prolong the growing season, which in turn promotes additional carbon sequestration and earlier plant growth (Nemani, 2003). Secondly, elevated temperatures can promote plant metabolism and net N mineralization, leading to an improved nutrient supply for the plant, this evidence was supported in our results (Figure 8). The GST exert great indirect effect on the ANPP and BNPP through affecting its influence on AN, which verify the hypothesis that the effects of air temperature on grassland productivity were mainly via altering soil available nitrogen. Furthermore, the effect of GST on ANPP was mainly through the influence of GST on grass ANPP rather than on other functional groups (Figure 6), suggesting that the grass group was more sensitive to changing temperature. Our results are supported by a previous study which found that elevated temperature could increase grass relative abundance, due to grasses having extensive, fibrous root systems that can exploit the increased N with under even a slight warming (Liu et al., 2018), in turn increasing grass ANPP. However, BNPP was not only determined by GST, but also by GSP (Figure 3). This is in contrast to ANPP, but similar to other studies that conducted in North and Central American grasslands (Hayes & Seastedt, 1987) where both temperature and precipitation were correlated significantly with BNPP. A potential explanation for the different controls of ANPP and BNPP could be the greater sensitivity of BNPP to precipitation owing to its greater need of dependence on water for growth. The water availability plays a vital role in nutrient mineralization and organic matter decomposition (Schimel & Parton, 1986), since all nutrients need to be dissolved in water in order to be available for absorption by roots. Thus, BNPP required more water to increase nutrient availability to plant roots or for transfer to the aboveground parts.

Furthermore, both ANPP and BNPP were controlled by AN, suggesting that the alpine *Kobresia* meadow was an N‐limited ecosystem (i.e., additional nitrogen addition could promote the productivity of grassland), which was consistent with other northern and temperate ecosystems (Bouwman, 2002). This suggested that the NPP might increase in future in the context of global warming and nitrogen deposition and that the enhanced temperature could further promote the mineralization of nitrogen. Overall, our study was based only on 8 years of NPP data, which might be too short to capture all characteristics of the temporal variations of NPP. Nevertheless, this long‐term field study provides very useful and valuable information to better understand the temporal variability of NPP, at least to get providing a more reliable quantification of NPP in an alpine ecosystem which is vulnerable and sensitive to climate change.

### 4.2 Dominant factors affecting the interannual variation of root turnover

Root turnover is a critical parameter of natural ecosystem, playing a vital role in carbon sequestration and nutrient dynamics (Gill & Jackson, 2000). Therefore, exploring the root turnover and it relationships with external conditions such as temperature and precipitation could yield a better understanding on how plant group response to climate change. A previous study shows the root turnover is largely controlled by temperature (Gill & Jackson, 2000), which is also supported by our observations that the root turnover was significantly positively related with GST (Figure 7). In addition, field observations and experimental manipulations indicated that the root lifespan decreased with temperatures (Mccormack & Guo, 2014), leading to a higher root turnover increased temperature. For instance, Tierney et al. (2001) found that the root turnover increased as root mortality increased, because removing snow increased temperature fluctuations. In general, a lower temperatures are often related to lower respiration rates because respiration rate (both autotrophic and heterotrophic) increases with temperature (Eissenstat, Wells, Yanai, & Whitbeck, 2000), thus enhanced temperature could increase root mortality by stimulating root physiological activities (Boone, Nadelhoffer, Canary, & Kaye, 1998). Furthermore, the elevated temperature could increase the nutrient availability for root, in turn further contributing to higher rates of root turnover through increasing root physiological activities (Gill & Burke, 2002). In contrast, the GSP exerts no significant effect on root turnover, which could attribute to the abundance soil water content during growing season.

### 4.3 Root turnover across different soil layer

A number of studies based on traditional coring techniques have concluded that the root turnover ratio of grassland ranged from 26%/year to 46%/year (Li, 1998) with an average of 34.7%/year (Jiyan & Yingnian, 2005). In this study, the root turnover ratio ranged from 26%/year to 41%/year from the soil surface to deep soil (i.e., from surface to 40 cm), with an average of 30%/year; such values are comparable to some previous reports (Jiyan & Yingnian, 2005) but lower than that of the global grass root turnover (53%/year) (Gill & Jackson, 2000). These discrepancies might arise from three factors. Firstly, the spatial scale was inconsistency, Gill and Jackson (2000) provide a global grass root turnover, whereas the calculation of root turnover in this study was based on local scale. Secondly, the methodological in calculating NPP is not consistent. At present, there are three methods of calculating NPP: max‐min, decision‐matrix, and positive increment methods, of which the max‐min and decision‐matrix methods often underestimate true production (Publicover & Vogt, 1993), and the apparent rate of root turnover at a single site could vary by an order of magnitude when using different estimation methods (Aber, Melillo, Nadelhoffer, Mcclaugherty, & Pastor, 1985). Therefore, it was not surprising to find differences of root turnover ratios in our results and other studies. Thirdly, other factors might also affect the root turnover rate such as environmental conditions, length of growing season, and nutrient supply (Nadelhoffer, 2000; Pregitzer, Hendrick, & Fogel, 1993). At a global scale, the average of root turnover rate is 53%/year, and exhibited a gradual decreasing trend from tropical to high‐latitude ecosystems (Gill & Jackson, 2000); this trend may be linked to climatic conditions. For instance, Ryser (1996) concluded that the root turnover ratio in poor nutrient ecosystems tended to reflect a longer root lifespan, in order to increase nutrient absorption, resulting in a lower root turnover rate than in nutrient‐rich ecosystems. Thus, we might attribute that the lower root turnover rate in our study to the nutrient‐poor environment and low temperature due to the low mineralization under low temperature in alpine ecosystem (Wu et al., 2011).

Interestingly, root turnover rate increased with soil depth, contrary to what would be expected. In general, the root turnover rate in deeper soil is lower than that in the surface soil layer because soil temperature, soil moisture, and nitrogen availability decreased with soil depth in the growing season (Hu et al., 2010). This pattern was not supported by controlling factors of root turnover in our study. For instance, the root turnover may also be strongly affected by root‐feeding herbivores, noting that the herbivore population increases with soil depth (Leetham & Milchunas, 1985), leading to a faster turnover rate of roots in deeper soil layers. An alternative explanation might be the different vertical distributions of root diameter class. Increasing evidence points to a root turnover rate that is largely determined by the diameter class of root (Richard A. Gill, Burke, Lauenroth, & Milchunas, 2002), with the fine roots (defined as ≤2 mm in diameter) often linked to relatively short longevity when compared with coarse roots (defined as >2 mm in diameter) due to their greater physiological activities such as higher root respiration rate and higher N concentration (Wells & Eissenstat, 2001). Furthermore, fine roots were mainly distributed in deeper soil layers in this alpine meadow, and decreased with soil depths, as supported by results of a previous study conducted at the same site (Wu et al., 2011).

## 5 CONCLUSIONS

Both ANPP and BNPP showed increasing trends during 2005–2015, with that of BNPP being significant. The BNPP decreased with soil depths; GST and GSP were significantly positively correlated with BNPP, but ANPP was only controlled by GST through its effect on grass ANPP. Furthermore, the AN strongly affected both ANPP and BNPP. The root turnover rate increased from shallower to deeper soil layers and was significant affected by GST and GSP. These results could enable better predictions of alpine ecosystem responses to climate change in the future.

## CONFLICT OF INTEREST

None declared.

## AUTHOR CONTRIBUTION

L Dai performed the research, analyzed data, and wrote the paper; F Zhang, X Guo, X Ke, Y Li, Y Du, C Peng, L Lin, Q Li and K Shu analyzed data; G Cao conceived the study.

## Data Availability

All data in this paper are available in Dryad: Dryad https://doi:10.5061/dryad.7sp252q.
